# Self-rated health, quality of life and appetite as predictors of initiation of dialysis and mortality in patients with chronic kidney disease stages 4–5: a prospective cohort study

**DOI:** 10.1186/s13104-018-3472-9

**Published:** 2018-06-08

**Authors:** Birgith Engelst Grove, Liv Marit Schougaard, Niels Henrik Hjollund, Per Ivarsen

**Affiliations:** 1 Department of Renal Medicine, Aarhus University Hospital & AmbuFlex/WestChronic, Regional Hospital West Jutland, Gl.Landevej 61, 7400 Herning, Denmark; 2 AmbuFlex/WestChronic, Regional Hospital West Jutland, Gl.Landevej 61, 7400 Herning, Denmark; 3 AmbuFlex/WestChronic, Occupational Medicine, University Research Clinic, Health, Aarhus University, Gl. Landevej 61, 7400 Herning, Denmark; 4 Department of Clinical Epidemiology, Aarhus University Hospital, Aarhus, Denmark; 5 Department of Renal Medicine, Aarhus University Hospital, Palle Juul-Jensens Boulevard 99, 8200 Aarhus N, Denmark

**Keywords:** Patient-reported outcome, Chronic kidney disease, Self-reported health, Quality of life, Pre-dialysis, Initiating dialysis, Transplantation, Mortality

## Abstract

**Objective:**

Patient-reported health status, including symptom burden, functional status and quality of life, are important measures of health in patients with chronic kidney disease. We aimed to investigate patient-reported outcomes (PRO) on self-rated health, appetite, quality of life and their associations with clinical outcomes. We conducted a prospective observational cohort study. Data was collected at baseline using a PRO questionnaire. The primary outcomes were initiation of dialysis, transplantation and mortality. Kaplan–Meier curves and multivariable Cox proportional hazards regression analyses were used.

**Results:**

A total of 126 patients with chronic kidney disease with an eGFR of ≤ 25 mL/min/1.73 m^2^ were followed for a median of 321 (range 10–523) days. Poor appetite was associated with mortality (hazard ratio 20.9, 95% CI 3.7–129.8). Initiation of dialysis was associated with low self-rated health (hazard ratio 5.2, 95% CI 1.2–24.0). Mean decline in estimated glomerular filtration rate was − 0.9 mL/min/1.73 m^2^ (95% CI − 1.6 to − 0.2). Decline in self-rated health (p = 0.001) and appetite (p = 0.002) were correlated with reduction in renal function.

**Electronic supplementary material:**

The online version of this article (10.1186/s13104-018-3472-9) contains supplementary material, which is available to authorized users.

## Introduction

Chronic kidney disease (CKD) is increasing globally as a consequence of people getting older, the increased prevalence of diabetes, hypertension and obesity and affects approximately 10% of the adult population [1]. Patients suffering from CKD need to modify their diet and lifestyle and attend regular medical appointments. A method to gain knowledge directly from the patients is through patient-reported outcome measures (PRO’s), which is defined as a measurement based on “any report of the status of a patient’s health condition that comes directly from the patient, without interpretation of the patient’s response by a clinician or anyone else” [2]. Use of PROs has increased in clinical practice with the recognition that measuring health as, for example, mortality and time to dialysis initiation, does not cover the complete perspective of the patient perception of health [3]. A recent comprehensive review concluded that use of PROs improves patient-centred care and supports clinical decisions [4]. Previous cohort studies in dialysis patients have suggested an association between self-reported health, quality of life (QoL), morbidity and mortality [5–7]. However, few studies have included patients prior to the onset of dialysis, and to the best of our knowledge none has investigated the relationship between PRO measures and renal function. The aim of this study was to investigate the association between PROs (self-rated health, appetite, quality of life) on initiation of dialysis, transplantation and mortality in patients with chronic kidney disease. We hypothesised that pre-dialysis patients with a low score in self-rated health or low QoL or poor appetite had an increased risk of death and initiation of dialysis during the follow-up period. Secondly, we aimed to describe the longitudinal changes in self-reported health and to compare these with changes in eGFR in a subgroup of patients’ not starting renal replacement therapy during follow-up.

## Main text

### Method

#### Study population

In total, 126 prevalent patients were included, in this prospective cohort study, from the outpatient clinic at Department of Renal Medicine, Aarhus University Hospital, Denmark from October 2013 to March 2014. Patients were eligible for inclusion if they were more than 18 years of age, had an eGFR of 25 mL/min/1.73 m^2^ or less and were candidates for active uraemia treatment. Exclusion criteria were cognitive impairment, visually impairment, incapable of using a touch screen or computer or having language difficulties. Patients were recruited in connection with their regular visits at the outpatient clinic, and sample size was based on feasibility rather than formal power calculations. A retrospective analysis revealed that the clinic in this inclusion period comprised 703 patients with an eGFR ≤ 25 mL/min. No significant differences were found between participants and non-participants with respect to age, gender and renal function (data not shown).

#### Data collection

The patients completed a questionnaire either at home or on a touch screen available at the clinic prior to each consultation; this was a part of daily routine clinical practice. The questionnaire consisted of a single item from SF-36 about self-rated health [8] and EQ-5D [9], which includes five multi-attribute items on QoL together with a kidney failure related question on self-rated appetite from the Kidney Disease and Quality of Life™ Short Form questionnaire [10]. The questionnaire was developed for clinical practice in collaboration between patients in the nephrology clinic and a group of experts consisting of physicians, a dietician, nurses working in the nephrology clinic and AmbuFlex. See Additional file 1: The Questionnaire. AmbuFlex is a web-administered PRO system and a method to use PRO in clinical practice where regularly scheduled follow-ups are substituted or supplemented with regular diagnosis-specific electronic questionnaires filled in by the patient prior consultation [11]. Staff nurses and the author (BEG) included patients consecutively. A graphical overview of the patients’ answers was available for the clinicians in the Electronic Medical Record (Additional file 2: Figure S1).

The endpoints were grouped into the four mutually exclusive categories: still in pre-dialysis care, in dialysis, transplanted and deceased (Table 2). Analyses of change in PRO measures in patients still in pre-dialysis at the end of follow-up included 42 patients. Time between filling in of the questionnaires was median 375 (IQR 322;488) days. 34 patients were lost to follow-up due to incomplete filling in of the questionnaire.

#### Variables

Clinical and demographic data were collected from medical records. Primary cause of kidney disease was classified using the ICD-10 codes (International Classification of Diseases) in accordance with previous national calculations [12]. Comorbidity was determined from chart reviews and categorised according to the Charlson Comorbidity Index (CCI) [13]. Laboratory measurements comprising estimated GFR calculated according to the MDRD 4-point formula [14]. Creatinine, albumin and haemoglobin were obtained from the Department of Clinical Biochemistry, Aarhus University Hospital. Blood samples were taken as part of routine practice at baseline and at follow-up, with an average time span from blood sampling to completion of the questionnaire of median 3 (range 1–7) days.

#### Statistical analyses

PRO data on appetite *“How would you rate your appetite?”* and health *“In general, would you say your health is?”* were categorised from five possible responses into three categories (*excellent/very good, good, fair/poor*). This was done because of the low incidences in the most extreme categories, as has been done in previous studies [5, 15]. Quality of life (QoL) was measured using the continuous EQ-5D score, which was categorised at the 25th and 75th percentile into three categories *‘very good’, ‘good’ and ‘low’* QoL in concordance with statistical recommendations. Baseline characteristics were presented for the total study population. Normally distributed variables were presented as mean ± standard deviation (SD), skewed distributed variables were presented as median with interquartile range (IQR). Kruskal–Wallis, *t* tests and X^2^ tests were used when appropriate. The analysis for the longitudinal change in PRO data was performed using the Wilcoxon signed-matched pair test. Kaplan–Meier curves were used to illustrate the proportion of participants reaching one of the endpoints during follow-up [16]. A Cox model adjusted for baseline covariates was estimated to determine the association between baseline PRO factors and dialysis, transplantation or mortality, with ‘*excellent/very good*’ as the reference category. *p* values of less than 0.05 were considered statistically significant. All analyses were performed in STATA statistical software, version 11.2.

### Results

#### Patient characteristics

Characteristics of the total study population are outlined in Table 1. At baseline, 35% of the study population rated their health as *poor*, 25% reported *low* QoL and 6% reported *poor* appetite. The majority of patients (60.3%) were still in pre-dialysis care, while the rest of the patents were almost equally distributed among the three other endpoint categories (Table 2).

**Table 1:** Baseline characteristics of study sample (N = 126) (Results/patient characteristics)

Characteristics	Total	
Age in years, median	68.5	[22]
Men, n (%)	83	(66)
Weight (kg)	76	[17]
BMI (kg/m^2^)	26	[6.5]
Systolic BP (mmHg)	138	[17]
Diastolic BP (mmHg)	78	[11]
eGFR (mL/min), median	14.5	[8]

Primary kidney disease^a^	N	%
Diabetes	23	(18)
Polycystic KD	11	(9)
Hypertension	9	(7)
Kidney transplanted	9	(7)
Glomerulonephritis	5	(4)
Vasculitis	4	(3)
Chronic interstitial nephritis	3	(2)
Other^b^	9	(7)
Unknown	53	(42)
*Medication intake*		
> 9 drugs per day	54	(43)
≤ 9 drugs per day	72	(57)
Use of phosphate binder	37	(29)
Use of erythropoietin	56	(44)
*Charlson Comorbidity Index (CCI)*		
Low (0–2)	50	(40)
Medium (3–5)	63	(50)
High (≥ 6)	13	(10)

*eGFR* estimated glomerular filtration rate, *BMI* body mass index

^a^ ICD-10 Classification

^b^ Obstructive, malignant, etc. [..] = IQR

**Table 2:** Baseline characteristics in 126 patients stratified by reached endpoint after follow-up

	Pre-dialyses patients N = 76^a^	Dialyses patients N = 21		Kidney transplanted N = 16		Deceased patients N = 13	
Characteristics	Median [IQR]	Median [IQR]	p value^b^	Median [IQR]	p value^c^	Median [IQR]	p value^d^
eGFR (mL/min/1.73 m^2^)	17 [8]	12 [4]	< 0.001	11.5 [3]	< 0.001	16 [6]	0.38
Serum albumin (g/L)	37 [6]^5^	34 [4]	0.03	38 [3]	0.9	36 [11]	0.31
CCI	3 [2]	4 [2]	0.02	2 [1]	0.73	4 [2]	0.06
Men, n (%)	44 (83)	16 (76)	0.13	14 (88)	0.03	9 (69)	0.44
Age, years	72 [17]	62 [15]	0.14	47 [16]	< 0.001	81 [10]	0.31
Drugs pEr day	8 [69]	10 [5]	0.02	8 [5]	0.40	10 [8]	0.28
Erythropoietin, n (%)	26 (34)	14 (67)	< 0.01	8 (50)	0.23	8 (24)	0.06
Phosphate binders, n (%)	17 (22)	10 (48)	0.03	6 (37)	0.21	4 (20)	0.51

Self-rated health	N (%)	N (%)		N (%)		N (%)	
Excellent/very good	19 (25)	2 (10)		4 (25)		1 (8)	
Good	38(50)	7 (33)		7 (44)		4 (31)	
Fair/poor	19(25)	12 (57)	0.02	5 (31)	0.65	8 (62)	0.03
*Self-rated appetite*							
Excellent/very good	54 (71)	9(43)		9 (56)		6 (46)	
Good	19 (25)	11(52)		7 (44)		4 (31)	
Fair/poor	3 (4)	1 (5)	0.05	–	0.78	3 (23)	0.03
*Quality of life*							
Very good	18 (24)	6 (29)		6 (38)		–	
Good	42 (55)	8 (38)		9 (56)		5(38)	
Low	16 (21)	7 (33)	0.34	1 (6)	0.05	8 (62)	0.01

*CCI* Charlson Comorbidity Index

^a^ Patients still in pre-dialysis care

^b^ Difference between dialysis starters and pre-dialysis group

^c^ Difference between the transplanted and pre-dialysis group

^d^ Difference between deceased and pre-dialysis group

^e^ 1 missing

#### Mortality

In total 13 (10.3%) of the patients died during follow-up. Compared to patients who remained in pre-dialysis, those who died during follow-up were more likely to rate their health as fair/poor, their appetite as fair/poor and their QoL as low (Table 2). Time until death is outlined in Kaplan–Meier curves, see Additional file 3: Figure S2. After adjustment for age, sex and comorbidity, only poor appetite remained significantly associated with increased risk of mortality (Table 3).

**Table 3:** Adjusted hazard ratio for mortality, initiating dialysis and transplantation associated with self-rated health, appetite and quality of life. N = 126

PRO	Health	Appetite	QoL
*Adjusted Hazard Ratio for mortality (n = 13)*			
Excellent/very good	1 (reference)	1 (reference)	1 (reference)
Good	0.98 (0.10–9.24)	0.91 (0.20–4.08)	0.35 (0.09–1.39)
Fair/poor	3.41 (0.41–28.1)	20.78* (3.46–124.71)	2.88 (0.71–11.58)
*Adjusted Hazard Ratio for initiation of dialysis (n = 21)*			
Excellent/very good	1 (reference)	1 (reference)	1 (reference)
Good	2.25 (0.46;11.1)	2.47 (0.99;6.23)	0.66 (0.23;1.98)
Fair/poor	5.18* (1.12;23.95)	1.59 (0.19;12.98)	1.22 (0.37;3.93)
*Adjusted Hazard Ratio for receiving a kidney transplant (n = 16)*			
Excellent/very good	1 (reference)	1 (reference)	1 (reference)
Good	1.68 (0.47;6.06)	1.37 (0.50;3.80)	1.16 (0.40;3.42)
Fair/poor	1.37 (0.34;5.51)	–	0.32 (0.03;3.43)

Numbers in table are Hazard Ratio (95% confidence interval). *QoL* quality of life

Adjusted for age, sex and comorbidity. * p < 0.05

#### Patients initiating dialysis

Twenty-one (16.7%) patients began dialysis during follow-up. Those who initiated dialysis during follow-up had a lower baseline eGFR compared to patients still in pre-dialysis care (*p* < 0.001) and a higher burden of comorbidity (*p* = 0.02) (Table 2). Patients with *poor* appetite, *low* QoL and *poor* self-rated health tended to initiate dialyses earlier, but no significant difference was found (Additional file 3: Figure S2). Patients reporting *fair/poor* health at baseline had an increased risk of initiating dialysis during follow-up (HR 5.18, 95% CI 1.12–23.95). Self-reported appetite or QoL did not associate with initiating dialysis (Table 3).

#### Transplantation

In total, 16 (13%) patients had a kidney transplant during follow-up. We found a shorter time to transplantation among the patients reporting *poor* health but no difference in appetite and QoL (Additional file 3: Figure S2). In the adjusted analysis we found no significant associations between self-rated health, appetite, QoL and transplantation (Table 3).

#### Change in renal function and the association with change in PRO data

A significant decline in eGFR (*p* = 0.02) from baseline until end of follow-up was found in patients (*n* = 76) still in pre-dialysis care. Mean decline in eGFR was − 0.89 mL/min/1.73 m^2^ (95% CI − 1.59 to − 0.19). A decline in eGFR was seen in 41 patients, 11 patients had an unchanged eGFR during follow-up, and eGFR increased in 24 patients. No significant differences were seen in serum albumin and haemoglobin (Additional file 4: Table S1). Patients with a decline in eGFR (*n* = 27) during follow-up reported an increased loss of appetite (*p* = 0.002) and decreased self-rated health (*p* = 0.001). QoL did not change. Among patients with a steady or an increased eGFR (*n* = 15), no significant difference in self-rated appetite, health or QoL was found. Adjustment for age, sex and comorbidity did not change the estimates (data not shown).

### Discussion

#### Main findings

In this prospective cohort study, poor appetite predicted increased risk of mortality and poor self-rated health was a predictor of initiation of dialyses in patients with CKD stage 4–5. We did not find any associations between PRO and transplantation. Decline in renal function was associated with decreased self-rated health and appetite.

#### Mortality

The mortality rate among pre-dialysis patients, was similar to previous findings reported in other pre-dialysis and dialysis populations [5, 17]. QoL was not associated with mortality. Previous studies have shown that QoL is a good indicator of physical well-being in a healthy elderly population as well as in patients on peritoneal dialysis, and QoL appears to be a strong predictor of morbidity and mortality [7, 18]. These studies used the SF-36 measurement tool and not the EQ-5D as done in present study. Recent data from the CRIC cohort including 3837 patients with CKD stages 2–4, with a follow-up time of 6.2 years demonstrated that low HRQoL, using the SF-36 measurement tool, was associated with increased risk of incident cardiovascular events and death but not CKD progression [19]. Interestingly, we found no significant association between self-rated health and mortality in present study. This may reflect our relatively small data set or the difference in follow-up time. In the adjusted analysis, in the present study, self-rated appetite was the only significant predictor of mortality, but confidence intervals were wide, probably due to a low number of events. In concordance with other studies, we also demonstrated that patients with poor appetite had low p-albumin, which in several previous studies has been shown to be a strong marker of mortality [15, 20, 21].

#### Initiating dialysis

Patients initiating dialysis reported a lower self-rated health compared to patients still in pre-dialysis care, which might reflect the burden of their symptoms related to the progression of renal failure. This finding concurs with a Japanese study among 471 patients with kidney failure, which showed a positive relation between loss of renal function and decline in several domains of SF-36 [22]. We found no association between QoL and initiation of dialysis. A similar study in a UK pre-dialysis population demonstrated a higher risk for death but not progression to End-Stage Renal Disease among those with a lower EQ-5D score [23].

#### Transplantation

We found no significant associations PRO measures and transplantation. The decision of transplantation relies mostly on availability of a suitable donor and therefore we did not have an a priori hypothesis concerning this endpoint. The negative result was therefore expected.

#### Change in renal function and the association with change in PRO data

Patients who had a decline in eGFR also experienced a decreased appetite and lower self-rated health. This indicates a relationship between eGFR and these PROs, which might be useful information to the clinicians when decision of future treatment is made together with the patient.

We found an association between self-reported low appetite and mortality during follow-up. Furthermore, low self-reported health was a predictor of initiating dialysis. In the context of the study limitations, our findings may indicate that implementing PRO in clinical practice could help identifying symptoms burden and prevent health impairment. Despite study limitations, the results of this study represent a step forward in exploring the use of PRO in a renal failure population. A randomised trial to investigate the implications of the use of PROs in this population has now been designed with a planned initiation of study enrolment September 2018.

## Limitations

This study had limitations such as a rather small sample size, as reflected by the wide confidence intervals. This limited our opportunity for statistical comparisons across disease stage and adjustment could only be performed for the most basic variables, thus residual confounding cannot be ruled out. This reduced our statistical power and accuracy of the results. The recruitment method and the relatively large number of non-participants may have introduced selection bias and thus reduced the external validity of our findings, however our drop out analysis showed no differences between participants and non-participants. The study was based only on a single institution, thus limiting the generalisability to other settings or different populations such as dialysis- or transplant patients. Accordingly, caution should be taken when interpreting our findings.

## Additional files

**Additional file 1.** The Questionnaire (Method/data collection).

**Additional file 2: Figure S1.** Graphical overview of patient reported outcome in the electronic medical journal (Method/data collection).

**Additional file 3: Figure S2.** Kaplan–Meier plot for initiation of dialysis, kidney transplantation and mortality (Results).

**Additional file 4: Table S1.** Laboratory values at baseline and after follow-up (N = 76) (Results/Change in renal function and the association with change in PRO data).

## Abbreviations

eGFR: estimated glomerular filtrations rate
QoL: quality of life
CCI: Charlson Comorbidity Index
ESRD: End Stage Renal Disease
CKD: chronic kidney disease
HRQoL: health related quality of life
