# Supplementary material for: Self-rated health, quality of life and appetite as predictors of initiation of dialysis and mortality in patients with chronic kidney disease stages 4–5: a prospective cohort study
Source: BMC Res Notes. 2018 Jun 8;11:371. doi: 10.1186/s13104-018-3472-9 (PMC5994035; doi:10.1186/s13104-018-3472-9)
Supplement: Supplementary file 1 — Additional file 1. The Questionnaire (Method/data collection). [file 13104_2018_3472_MOESM1_ESM.pdf]

YOUR KIDNEY DISEASE

During the past 4 weeks, to what extent were you bothered by each of the following?

Nausea	☐ Not at all	☐ Somewhat	☐ Moderately	☐ Very much	☐ Extremely
Vomiting	☐ Not at all	☐ Somewhat	☐ Moderately	☐ Very much	☐ Extremely
Itchy skin	☐ Not at all	☐ Somewhat	☐ Moderately	☐ Very much	☐ Extremely
Shortness of breath	☐ Not at all	☐ Somewhat	☐ Moderately	☐ Very much	☐ Extremely
Thirst	☐ Not at all	☐ Somewhat	☐ Moderately	☐ Very much	☐ Extremely
Swollen legs	☐ Not at all	☐ Somewhat	☐ Moderately	☐ Very much	☐ Extremely
Leg cramps	☐ Not at all	☐ Somewhat	☐ Moderately	☐ Very much	☐ Extremely
Dizziness	☐ Not at all	☐ Somewhat	☐ Moderately	☐ Very much	☐ Extremely
Difficulty concentrating	☐ Not at all	☐ Somewhat	☐ Moderately	☐ Very much	☐ Extremely
Washed out or drained	☐ Not at all	☐ Somewhat	☐ Moderately	☐ Very much	☐ Extremely
Difficulty remembering	☐ Not at all	☐ Somewhat	☐ Moderately	☐ Very much	☐ Extremely
Feeling tired	☐ Not at all	☐ Somewhat	☐ Moderately	☐ Very much	☐ Extremely
Nervousness or restlessness	☐ Not at all	☐ Somewhat	☐ Moderately	☐ Very much	☐ Extremely
Feeling annoyed or irritated	☐ Not at all	☐ Somewhat	☐ Moderately	☐ Very much	☐ Extremely
Trouble sleeping at night	☐ Not at all	☐ Somewhat	☐ Moderately	☐ Very much	☐ Extremely
Worried about the future	☐ Not at all	☐ Somewhat	☐ Moderately	☐ Very much	☐ Extremely

QUALITY OF LIFE

Appetite

How would you rate your appetite?	☐ Excellent	☐ Very good	☐ Good	☐ Fair	☐ Poor
How many meals do you eat a day?	☐ 1 - 2	☐ 3	☐ 4	☐ 5	☐ More than 5
Has your appetite changed within the last 4 weeks?	☐ It's become much bigger	☐ It's become a bit bigger	☐ It's unchanged	☐ It's become a bit smaller	☐ It's become a lot smaller
Does food taste differently now than before?	☐ No	☐ Yes, a little	☐ Yes, somewhat	☐ Yes, a lot	☐ Don't remember

General health

In general, would you say your health is:	☐ Excellent	☐ Very good	☐ Good	☐ Fair	☐ Poor
Compared to one year ago, how would you rate your health in general now?	☐ Much better now than one year ago	☐ Somewhat better now than one year ago	☐ About the same as one year ago	☐ Somewhat worse now than one year ago	☐ Much worse now than one year ago

Under each heading, please tick the ONE box that best describes your health TODAY

MOBILITY	☐ I have no problems in walking about	☐ I have slight problems in walking about	☐ I have moderate problems in walking about	☐ I have severe problems in walking about	☐ I am unable to walk about
SELF-CARE	☐ I have no problems washing or dressing myself	☐ I have slight problems washing or dressing myself	☐ I have moderate problems washing or dressing myself	☐ I have severe problems washing or dressing myself	☐ I am unable to wash or dress myself
USUAL ACTIVITIES (e.g. work, study, housework, family or leisure activities)	☐ I have no problems doing my usual activities	☐ I have slight problems doing my usual activities	☐ I have moderate problems doing my usual activities	☐ I have severe problems doing my usual activities	☐ I am unable to do my usual activites
PAIN / DISCOMFORT	☐ I have no pain or discomfort	☐ I have slight pain or discomfort	☐ I have moderate pain or discomfort	☐ I have severe pain or discomfort	☐ I have extreme pain or discomfort
ANXIETY / DEPRESSION	☐ I am not anxious or depressed	☐ I am slightly anxious or depressed	☐ I am moderately anxious or depressed	☐ I am severely anxious or depressed	☐ I am extremely anxious or depressed
