# Supplementary material for: Self-rated health, quality of life and appetite as predictors of initiation of dialysis and mortality in patients with chronic kidney disease stages 4–5: a prospective cohort study
Source: BMC Res Notes. 2018 Jun 8;11:371. doi: 10.1186/s13104-018-3472-9 (PMC5994035; doi:10.1186/s13104-018-3472-9)

**Additional file 2**

**Figure S1. Graphical overview of patient reported outcome in the electronic medical journal**


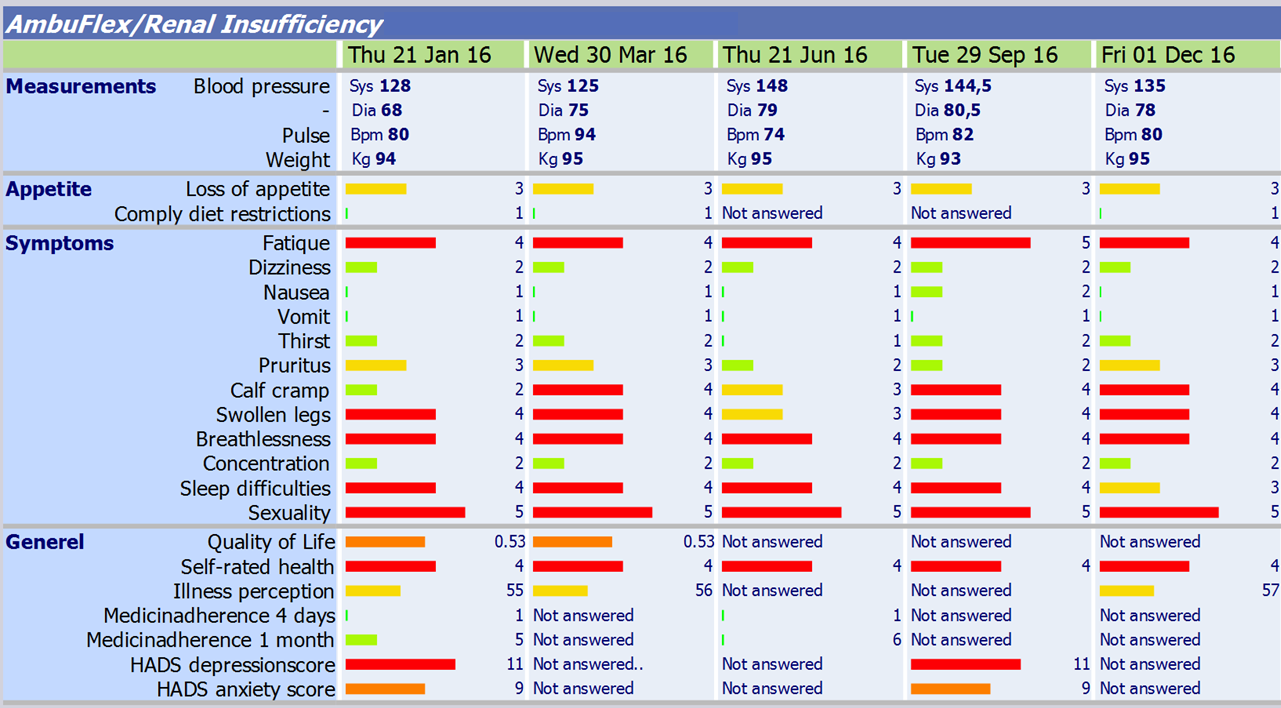

Supplement: Supplementary file 2 — Additional file 2: Figure S1. Graphical overview of patient reported outcome in the electronic medical journal (Method/data collection). [file 13104_2018_3472_MOESM2_ESM.docx]
