# Supplementary figures and images for: Self-rated health, quality of life and appetite as predictors of initiation of dialysis and mortality in patients with chronic kidney disease stages 4–5: a prospective cohort study
Source: BMC Res Notes. 2018 Jun 8;11:371. doi: 10.1186/s13104-018-3472-9 (PMC5994035; doi:10.1186/s13104-018-3472-9)

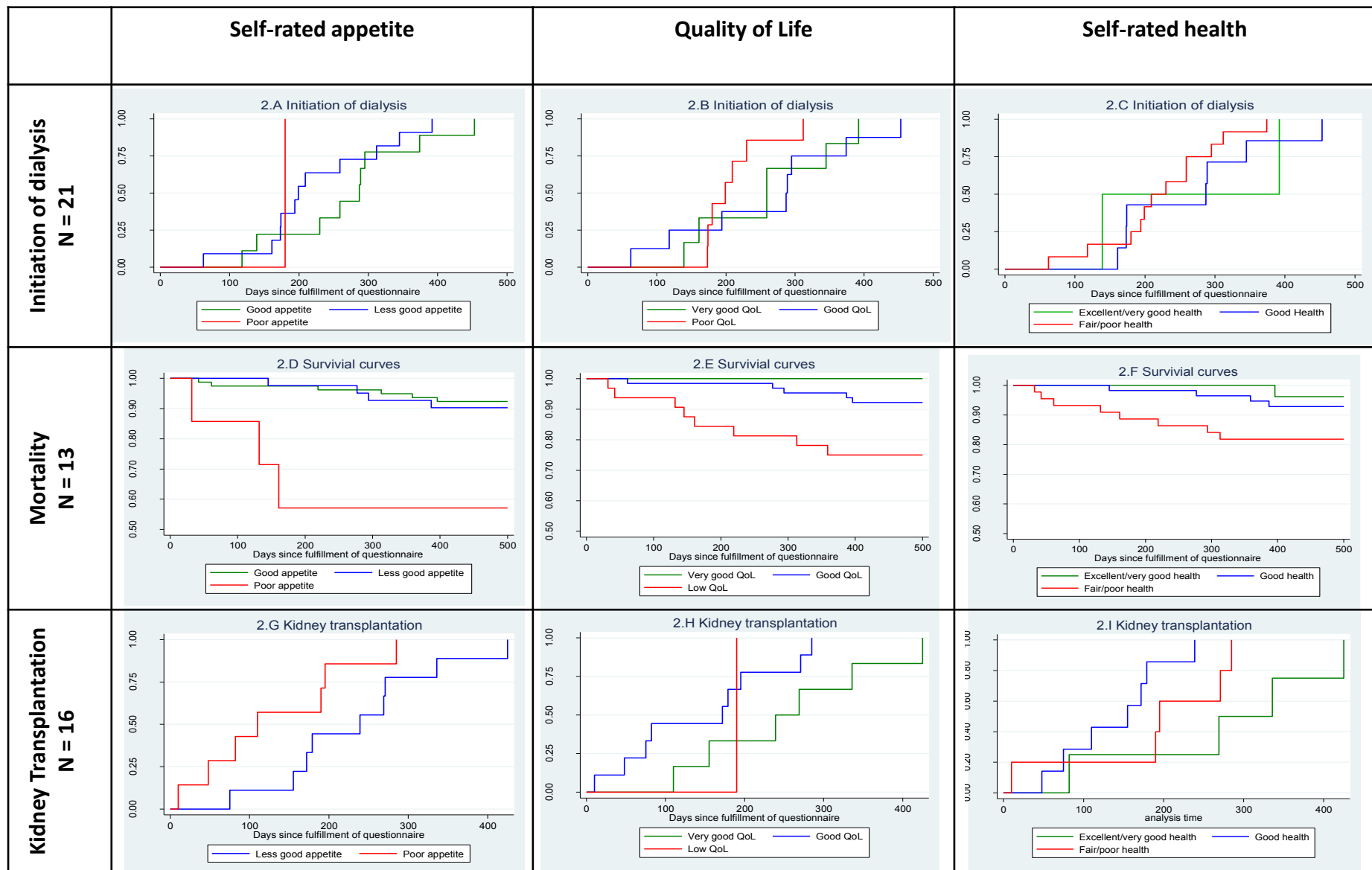

Supplement: Supplementary file 3 — Additional file 3: Figure S2. Kaplan–Meier plot for initiation of dialysis, kidney transplantation and mortality (Results). [file 13104_2018_3472_MOESM3_ESM.pdf]
