# Supplementary material for: Self-rated health, quality of life and appetite as predictors of initiation of dialysis and mortality in patients with chronic kidney disease stages 4–5: a prospective cohort study
Source: BMC Res Notes. 2018 Jun 8;11:371. doi: 10.1186/s13104-018-3472-9 (PMC5994035; doi:10.1186/s13104-018-3472-9)
Supplement: Supplementary file 4 — Additional file 4: Table S1. Laboratory values at baseline and after follow-up (N = 76) (Results/Change in renal function and the association with change in PRO data). [file 13104_2018_3472_MOESM4_ESM.docx]

Additional file 4

(Results/Change in renal function and the association with change in PRO data)

Table S1. Laboratory values at baseline and after follow-up (N = 76)

|  | **Baseline** | | | **Follow-up** | | |  |
| --- | --- | --- | --- | --- | --- | --- | --- |
| **Blood-samples** | **n** | **Median** | **IQR** | **n** | **Median** | **IQR** | **p.value*^1^** |
| S- creatinine (µmol/l) | 76 | 288 | 176.5 | 75¤ | 307 | 201 | 0.03 |
| S –albumin (g/L) | 75 | 37 | 6 | 74¤ | 36 | 7 | \| 0.22 \| \| --- \| |
| eGFR (mL/min/1.73m^2^) | 76 | 17 | 8.5 | 75¤ | 16 | 10 | 0.02 |
| S-haemoglobin (µmol/L) | 75 | 7.5 | 1.2 | 75 | 7.3 | 0.90 | 0.23 |
| *Wilcoxon signed rank-sum test used, 1) p value symbols a significant difference between the 76 persons at baseline and follow-up. ¤ 1 person missing | | | | | | | |
